# Neutrophil infiltration and myocarditis in patients with severe COVID-19: A post-mortem study

**DOI:** 10.3389/fcvm.2022.1026866

**Published:** 2022-10-14

**Authors:** Quanyu Zhang, Huarong Zhang, Xiaowei Yan, Sicong Ma, Xiaohong Yao, Yu Shi, Yifang Ping, Mianfu Cao, Chengfei Peng, Shuai Wang, Min Luo, Chenghui Yan, Shuyang Zhang, Yaling Han, Xiuwu Bian

**Affiliations:** ^1^Department of Cardiology, General Hospital of Northern Theater Command, Shenyang, China; ^2^Institute of Pathology and Southwest Cancer Center, Southwest Hospital, Third Military Medical University (Army Medical University), Beijing, China; ^3^Division of Cardiology, Peking Union Medical College (PUMC) Hospital, PUMC & Chinese Academy of Medical Sciences, Beijing, China

**Keywords:** COVID-19, autopsy, heart, myocarditis, neutrophil infiltration

## Abstract

**Aims:**

To investigate cardiac pathology in critically ill patients with coronavirus disease 2019 (COVID-19) and identify associations between pathological changes and clinical characteristics.

**Methods:**

The present autopsy cohort study included hearts from 26 deceased patients hospitalized in intensive care units due to COVID-19, and was conducted at four sites in Wuhan, China. Cases were divided into a neutrophil infiltration group and a no-neutrophil group based on the presence or absence of histopathologically identified neutrophilic infiltrates.

**Results:**

Among the 26 patients, histopathological examination identified active myocarditis in four patients. All patients with myocarditis exhibited extensive accompanying neutrophil infiltration, and all patients without myocarditis did not. The neutrophil infiltration group exhibited significantly higher rates of detection of interleukin-6 (100 vs. 4.6%) and tumor necrosis factor-alpha (100 vs. 31.8%) than the no-neutrophil group (both *p* < 0.05). On admission, four patients with neutrophil infiltration in myocardium had significantly higher baseline levels of aspartate aminotransferase, D dimer, and high-sensitivity C reactive protein than the other 22 patients (all *p* < 0.05). During hospitalization, patients with neutrophil infiltration had significantly higher maximum creatine kinase-MB (median 280.0 IU/L vs. 38.7 IU/L, *p* = 0.04) and higher troponin I (median 1.112 ng/ml vs. 0.220 ng/ml, *p* = 0.56) than patients without neutrophil infiltration.

**Conclusion:**

Active myocarditis was frequently associated with neutrophil infiltration in the hearts of deceased patients with severe COVID-19. Patients with neutrophil-infiltrated myocarditis had a series of severely abnormal laboratory test results on admission, and high maximum creatine kinase-MB during hospitalization. The role of neutrophils in severe heart injury and systemic conditions in patients with COVID-19 should be emphasized.

## Introduction

Coronavirus disease 2019 (COVID-19) outbreaks caused by severe acute respiratory syndrome coronavirus 2 (SARS-CoV-2) still occur repeatedly and intermittently around the world. Although COVID-19 is mainly characterized by the infection of the lung and respiratory failure, cardiac injury with troponin elevation is evidently associated with mortality (1, 2). In several post-mortem autopsy studies, heart tissue and cardiomyocyte injury including myocardial necrosis were common and non-specific, but the rate of pathology-confirmed myocarditis was low (3, 4). Despite being found in heart tissue, the presence of SARS-CoV-2 as determined via reverse transcription-polymerase chain reaction (RT-PCR) was rarely detected in cardiomyocytes; thus, it was unclear whether direct virus invasion was the primary cause of cardiac injury (5, 6). To date, the precise mechanisms involved in pathological changes in the heart induced by COVID-19 are unclear.

A proportion of patients with COVID-19 progress to critical illness, and are at significantly higher risk of mortality (7, 8). Especially with the current rapid spread of Delta and Omicron variants, an increasing trend of severe cases with worse prognoses has emerged (9). Critically ill patients experience a long stay in the intensive care unit (ICU), and are more prone to developing multiple organ dysfunction syndromes including the heart (10, 11), which may result in substantial histological and immunological changes. Therefore, we conducted a post-mortem pathological study of critically ill patients with COVID-19 to investigate pathological features of hearts and associations between pathological changes and clinical characteristics.

## Materials and methods

### Study population and specimen disposal

This autopsy cohort study included 26 patients with COVID-19 from Huoshenshan Hospital (*n* = 8), Taikang Tongji Hospital (*n* = 5), Zhongfaxincheng Hospital (*n* = 5), and Wuhan Jinyintan Hospital (*n* = 8), China, who died between 18 February 2020 and 04 April 2020. Patient hospitalization information has been described previously (12). Briefly, all 26 patients had COVID-19 confirmed via nasopharyngeal or pharyngeal PCR analyses of SARS-CoV-2 RNA and were hospitalized in the ICU. Full autopsies were performed with the approval of the relevant ethics committees, and written consent from the patient's relatives in accordance with regulations issued by the National Health Commission of China and the Helsinki Declaration.

Clinical characteristics, laboratory tests, echocardiography results, complications during hospitalization, medications, and invasive procedures undertaken were ascertained from hospitalization records and other sources of information. Time from syndrome onset to hospitalization was also recorded. For laboratory tests including cardiac markers and inflammatory indicators, baseline values on admission and maximum values during hospitalization were recorded. Laboratory tests include creatine kinase (CK), creatine kinase-MB (CK-MB), hypersensitive troponin I (hsTnI), brain natriuretic peptide (BNP), interleukin (IL) 6, hypersensitive C reactive protein (hsCRP), procalcitonin (PCT), alanine aminotransferase (ALT), and aspartate aminotransferase (AST) were measured at a core laboratory within each participating site within minutes after blood drainage. Serum samples were then centrifuged, and serum was separated and stored at −80°C for repeated measurements if necessary. To minimize autolysis, decedents were promptly stored at 4°C after death and the range of the post-mortem interval (time of death to time of autopsy) was 4–24 h. For histopathological analysis, autopsy materials were collected, fixed in 4% neutral formaldehyde for at least 24 h, formalin-fixed, and embedded in paraffin.

### Pathological analysis

Autopsies of hearts were performed by two experienced pathologists, and ventricle tissues, atrium tissues, and epicardial coronary arteries were collected for further analyses. A median of 25 full-thickness blocks of myocardium was examined histologically (range 11–40 blocks). Pathological changes in hearts were evaluated via hematoxylin and eosin (H&E) staining and immunohistochemical (IHC) staining. H&E staining was performed in accordance with a standard procedure. IHC staining was performed using routine automated diagnostic IHC staining devices (Roche, BenchMark-ultra). Myocarditis was defined as microscopic findings of multiple foci of increased leukocyte infiltration associated with myocyte injury that was not due to another cause (3). The number of myocardium-infiltrating mononuclear cells per mm^2^ in a high-power field was counted in each sample with the most inflammation, using IHC staining for CD4 (Zhongshan Jinqiao, #ZM-0418), CD8 (Zhongshan Jinqiao, #ZA-0508), CD20 (Zhongshan Jinqiao, #ZM-0039), and CD68 (Zhongshan Jinqiao, #ZM-0060). Primary antibodies used for IHC staining included IL-6 (Abcam, ab6672, 1:600) and tumor necrosis factor-alpha (TNF-α; Cell Signal Technology, #8184, 1:20). Images were captured using a digital camera (DP73, Olympus) under a light microscope (BX43, Olympus). The diluent without primary antibodies was used as a negative control for IHC staining.

### Statistical analysis

Continuous variables are presented as means ± the standard deviation (SD) or medians with ranges for non-parametric data. Categorical data are presented as counts with percentages. To quantify correlations between pathological findings and clinical characteristics, the Kendall's tau-b index for bivariate correlational analysis was used. Two-sided *p*-values < 0.05 were considered statistically significant. All statistical analyses were performed via IBM SPSS version 25 and R version 3.6.1.

## Results

A total of 26 patients admitted to ICUs due to COVID-19 were included in this pathological study. General patient characteristics and main causes of death have been published previously (12). Briefly, the median age of the study cohort was 68 years (range 53–88 years), and 50% (13 patients) were male subjects. The median duration in the ICU until death was 20 days (range 3–61 days). Twenty patients had at least one comorbidity, including 10 with chronic cardiovascular diseases (three with coronary artery disease, three with cardiac dysfunction, two with valvular heart disease, one with dilated cardiomyopathy, and one with arrhythmia), nine with hypertension, six with chronic pulmonary diseases, and four with diabetes. Most of the 26 patients died of pulmonary injuries related to COVID-19.

Heart failure occurred in 10 (38.5%) patients. Atrial fibrillation was documented in six (23.1%) patients and was the main type of new-onset arrhythmia during hospitalization. Due to their serious illness, various complications emerged during hospitalization in the ICU including respiratory failure, pleural effusion, pneumothorax, anemia, renal dysfunction, and disseminated intravascular coagulation. Eighteen patients received anticoagulation treatment, and two received antiplatelet therapy. Multiple invasive procedures including non–end-stage endotracheal intubation, assisted ventilation, deep vein puncture, bronchoscopy, dialysis, and extracorporeal membrane oxygenation were intermittently or continuously used in critical situations. Treatment information is shown in Supplementary Table S1.

### Pathological findings

**Figure 1:**
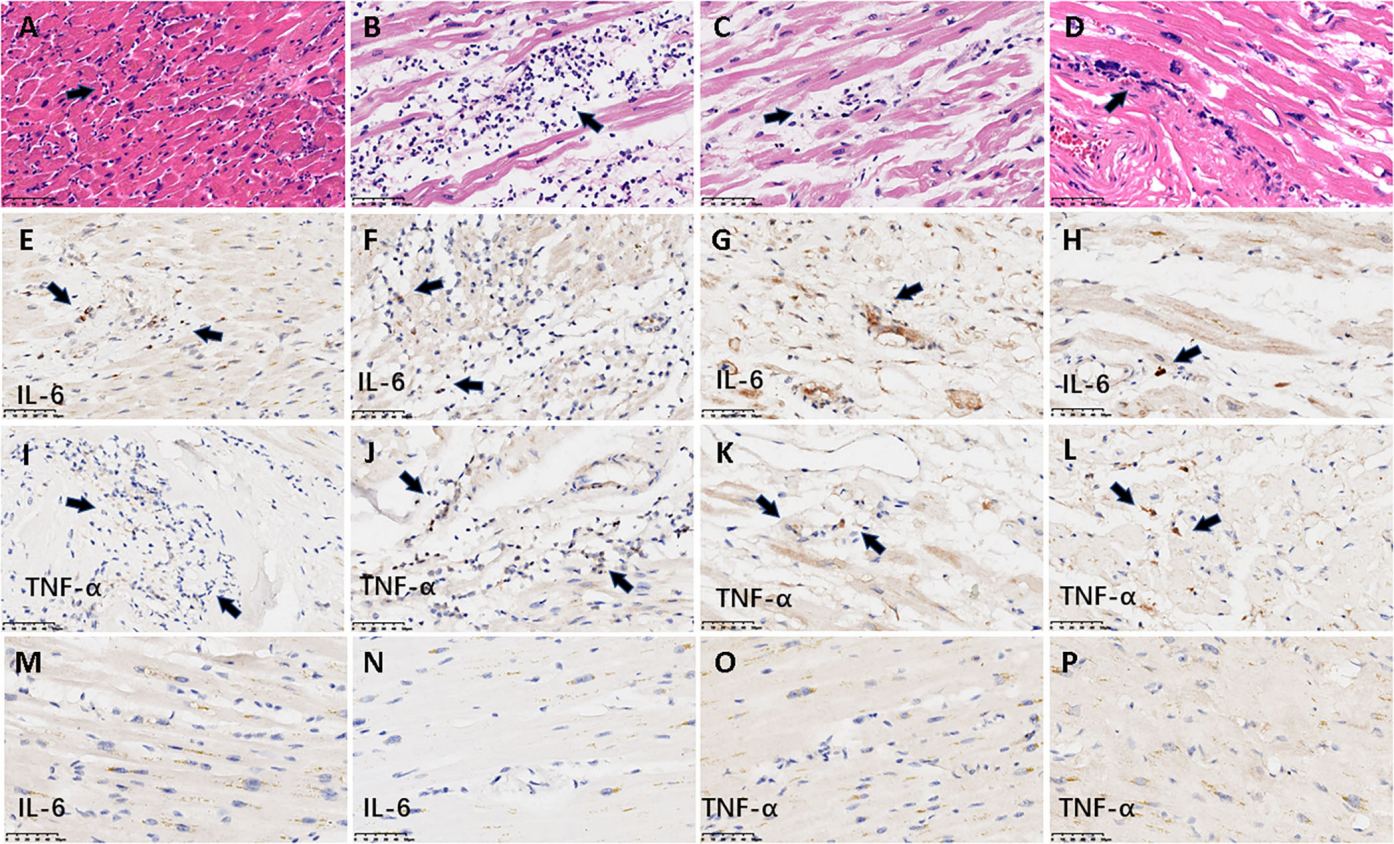
Representative histological and IHC findings from hearts. The figure shows the histological and IHC findings from heart tissues. **(A–D)** demonstrate active myocarditis in the four cases. **(A,B)** The histology in the myocardium demonstrated diffuse neutrophilic infiltrates with myocyte injury in a 62-year-old man and a 56-year-old woman, respectively; **(C,D)** multiple small discrete foci of mixed inflammatory cells with visible neutrophils associated with single-cell necrosis of cardiomyocytes in a 63-year-old woman and a 76-year-old man, respectively. The arrows denote the infiltrated neutrophils. **(E–L)** denote the positive IHC staining of IL-6 and TNF-α from the four cases with active myocarditis. **(E–H)** IL-6; **(I–L)** TNF-α. The longitudinal images in the first three rows were derived from the same case (A, E, I from a 62-year-old man; B, F, J from a 56-year-old woman; C, G, K from a 63-year-old woman; D, H, L from a 76-year-old man). Arrows denote the positive signal of IHC staining. **(M–P)** Represent the negative expression of IL-6 and TNF-α from two cases without neutrophil infiltration. **(M,O)** IHC staining from an 81-year-old man; **(N,P)** IHC staining from a 59-year-old woman. Scale bars represent 50μm. IHC: immunohistochemical.

As described in our previous work, a series of common pathological changes in hearts were found in all 26 patients, including myocardial cell degeneration and scattered necrosis, mild interstitial edema, and infiltration of monocytes and lymphocytes and/or neutrophils (13). Cardiomyocyte hypertrophy, atrophy, and interstitial fibrosis of varying degrees based on underlying diseases were also detected. Morphological analysis of heart tissue blocks identified active myocarditis in only four (15.4%) patients (**Figure 3**). Neutrophilic infiltrates were detected in all four patients with myocarditis. Diffuse neutrophilic infiltrates were associated with adjacent cardiomyocyte degeneration or necrosis, and involvement of bilateral ventricles and atriums was detected in two of the patients with myocarditis. In one of these patients, there was obvious accompanying myocardial interstitial edema (Figures 1A,B). In the two other patients with active myocarditis, there were multiple small discrete foci of mixed inflammatory cells with visible neutrophils and lymphocytes associated with single-cell necrosis of cardiomyocytes, involving the left ventricle and atrium (Figures 1C,D). All 22 patients without active myocarditis exhibited minor infiltration of scattered mononuclear cells in the myocardial interstitium, rather than neutrophils. To further investigate the severity and properties of inflammation in myocardium, IHC staining was performed to detect the expression of TNF-α and IL-6. Expression of TNF-α and IL-6 in infiltrating inflammatory cells and myocardial interstitial cells was detected in all four patients with neutrophil infiltration (Figures 1E–L), whereas patients without neutrophil infiltration exhibited negative or mild expression of these inflammation-related factors (Figures 1M–P). Patients with neutrophil infiltration were more likely to exhibit TNF-α and IL-6 positivity than those without neutrophil infiltration [TNF-α 100 vs. 31.8% (7/22 cases), *p* = 0.022; IL-6 100% vs. 4.6 (1/22 cases), *p* < 0.001; Table 1].

**Table 1:** Pathologic findings of cases with vs. without neutrophil infiltration.

**Pathologic findings**	**Cases with neutrophil infiltration (myocarditis) N = 4**	**Cases without neutrophil infiltration (myocarditis) N = 22**	**P value**
**TNF-α** **(+), No. (%)**	4 (100%)	7 (32%)	0.02
**IL-6 (+), No. (%)**	4 (100%)	1 (5%)	< 0.001
**Number of lymphocytes per mm** ^**2**^ **, median (range)**
CD4+ cell	6.5 (range 4–13)	8.5 (range 3–17)	0.45
CD8+ cell	8 (range 5–15)	12 (range 3–50)	0.29
CD20+ cell	2 (range 1–5)	3 (range 1–5)	1.00
CD68+ cell	61 (range 34–89)	50 (range 24–154)	0.83
**Microthrombi (+), No. (%)**	4 (100%)	8 (36%)	0.02

TNF-α, tumor necrosis factor; IL-6, interleukin-6.

**Figure 2:**
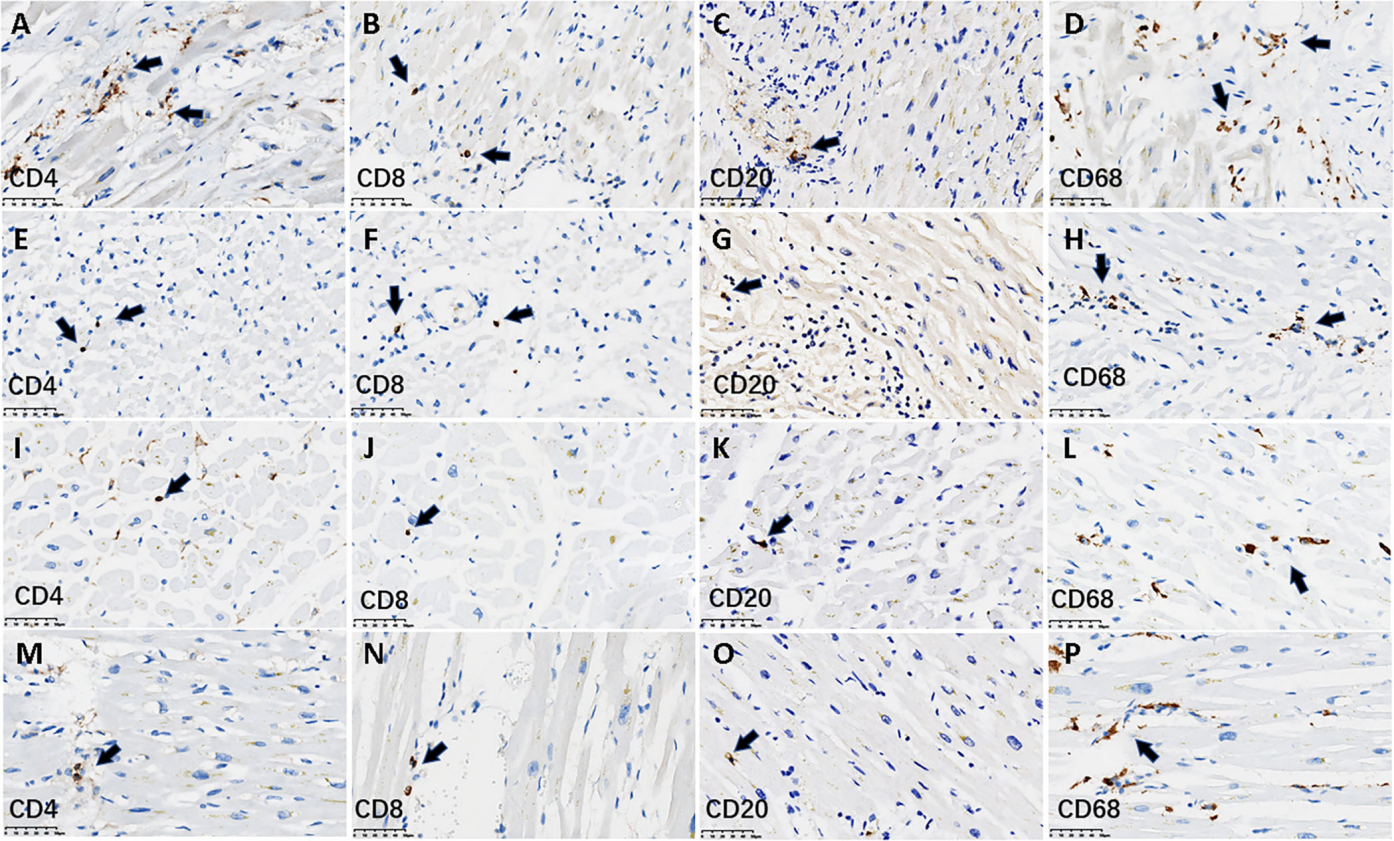
Lymphocyte infiltration using IHC staining within myocardium in representative cases. The figure shows the infiltration of lymphocytes and macrophages stratified by CD4^+^, CD8^+^, CD20^+^, and CD68^+^ cells in the myocardium in four cases. **(A–H)** denote IHC staining for lymphocytes from two cases with neutrophil-infiltrated pathological myocarditis. **(A–D)** 62-year-old man; **(E–H)** a 56-year-old woman. **(I–P)** denote IHC staining for lymphocytes from two cases without pathological myocarditis. **(I–L)** An 81-year-old man; **(M–P)** a 59-year-old woman. The immunostaining of two myocarditis cases showed there was a scattered infiltration of very mild CD4^+^, CD8^+^, CD20^+^ lymphocytes, and single or small clusters of CD68+ macrophages. This pattern was also seen in the other two cases without myocarditis. The arrows denote the lymphocytes or macrophages. Scale bars represent 50 μm. IHC, immunohistochemical.

The immunologic characteristics of myocardium-infiltrating mononuclear cells were analyzed using IHC staining for the helper T cell marker CD4, the cytotoxic T cell marker CD8, the B cell marker CD20, and the monocyte and macrophage marker CD68. All four patients with active myocarditis exhibited very mild infiltration of CD4^+^, CD8^+^, and CD20^+^ lymphocytes, and single or small clusters of CD68^+^ macrophages, and this pattern was also evident in all 22 patients without active myocarditis (Figure 2). The numbers of each cell type per mm^2^ in a high-power field are shown in Table 1. There were no significant differences in CD4^+^, CD8^+^, CD20^+^, or CD68^+^ cell densities between patients with and without active myocarditis (all *p* > 0.05; Table 1). Kendall's tau-b index indicated no significant correlations between the numbers of each cell type and the levels of TNF-α or IL-6 expression (all *p* > 0.05). Other pathologic findings are shown in Supplementary Figure S1. Two of the four patients with neutrophil infiltration had neutrophil-predominant endocarditis. Dilated cardiomyopathy with tricuspid valve infective endocarditis occurred in one patient without neutrophil infiltration. Epicarditis with focal infiltration of mixed inflammatory cells occurred in three patients with neutrophil infiltration and seven patients without neutrophil infiltration. Mixed thrombi were detected in four patients without neutrophil infiltration, including one in the left atrium, one in the right atrium, and two in the right ventricle. Epicardial coronary arteriosclerosis was detected in nine of the 26 patients, including one with neutrophil infiltration. There was no thrombotic occlusion or endarteritis of the epicardial coronary artery in any of the 26 patients. Intravascular microthrombi in myocardial interstitium were observed via microscopy in 12 (46.2%) patients, including all four with neutrophil infiltration and another eight without neutrophil infiltration. The detection rate of cardiac microthrombi in patients with neutrophil infiltration was significantly higher than that in patients without neutrophil infiltration (100% vs. 36.4%, *p* = 0.02; Table 1).

### Clinical characteristics of the neutrophil infiltration and no-neutrophil infiltration groups

**Figure 3:**
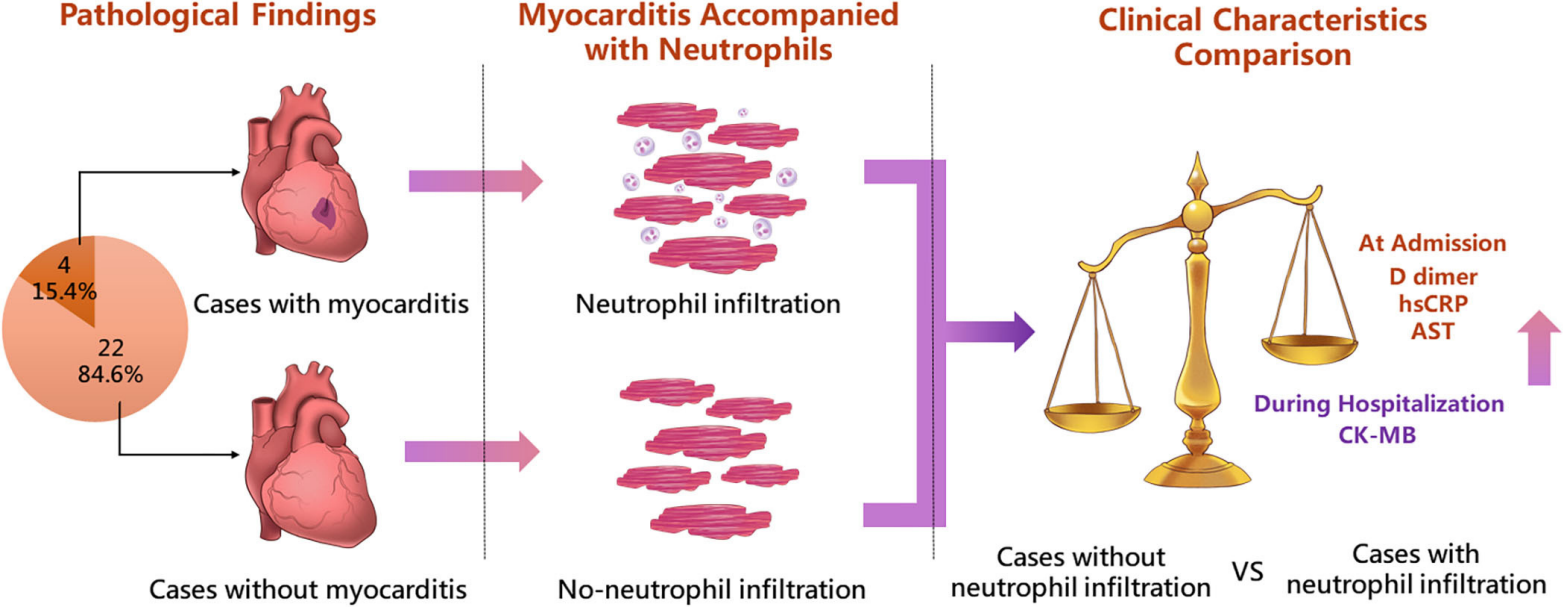
Highlight overview. The figure shows the highlight overview of the present autopsy cohort study. Neutrophil infiltration in heart tissue was associated with active pathological myocarditis in COVID-19. Patients with neutrophil infiltration had dramatically elevated levels of both cardiac and systemic laboratory tests, indicating the severe condition of COVID-19. hsCRP, high-sensitivity C reactive protein; AST, aspartate aminotransferase.

To investigate dynamic changes in clinical characteristics in the 26 deceased patients, baseline characteristics at admission, and maximum values of a series of laboratory test parameters reflecting severe medical conditions were analyzed. Baseline characteristics including parameters of cardiac injury, inflammation, coagulation, and liver function at admission are shown in Table 2. The median time from symptom onset to hospital admission in patients with neutrophil infiltration was 20.5 days (range 13–26 days), which was significantly longer than that in patients without neutrophil infiltration (10.0 days, range 1–24 days; *p* = 0.02). Compared to patients without neutrophil infiltration, those with neutrophil infiltration had significantly higher baseline levels of AST, D dimer, and hsCRP (all *p* < 0.05, Table 2, Figure 3). In terms of baseline cardiac markers, median CK was significantly higher in patients with neutrophil infiltration than in those without neutrophil infiltration (277.5 IU/L, range 91.0–486.0 IU/L vs. 36.0 IU/L, range 11.5–547.0 IU/L; *p* = 0.03). CK-MB and BNP were similar in the two groups (both *p* > 0.05; Table 2, Figure 3). Baseline hsTnI was only available for 16 (61.5%) of the 26 patients. In the other 10 (38.5%) patients, hsTnI was not evaluated until they were transferred to the ICU (*n* = 6) or exhibited symptoms indicating heart failure or atrial fibrillation (*n* = 4).

**Table 2:** Clinical characteristics of cases with vs. without neutrophil infiltration.

	**Neutrophil (–) *N* = 22**	**Neutrophil (+) *N* = 4**	**P value**	**Neutrophil (–) *N* = 22**	**Neutrophil (+) *N* = 4**	**P value**
Age, years, (median, range)	69 (53–88)	62.5 (56–77)	0.21			
Sex, male, No. (%)	11 (50.00%)	2 (50.00%)	1.00			
Time from symptom onset to hospital admission, days, (median, range)	10 (1–24)	20.5 (10–26)	0.01			
**Laboratory test (median, range)**	**Baseline characteristics at admission**			**Maximum value during hospitalization**		
CK-MB, IU/L	11.4 (5.5–45.0)	13.5 (7.20–128.0)	1.00	38.7 (5.9–234.7)	280.0 (14.0–996.0)	0.04
CK, IU/L	36.0 (11.5–547.0)	277.5 (91.0–486.0)	0.03	144.9 (36.0–1,997.0)	2,276.5 (91.0–7,491.0)	0.17
hsTnI, ng/ml	NA	NA	NA	0.220 (0.008–8.749)	1.112 (0.008–7.775)	0.56
BNP, pg/ml	55.1 (10.0–1,243.0)	56.7 (10.0–299.1)	0.80	387.0 (89.3–26,000.0)	173.0 (56.0–328.0)	0.19
IL-6, pg/ml	6.89 (16.80–204.10)	16.54 (6.89–26.30)	0.48	63.44 (16.14–5,000.00)	20.28 (11.79–455.00)	0.19
hsCRP, mg/L	10.0 (2.15–160)	96.6 (67.6–211.0)	0.02	10.0 (9.5–163.5)	134.0 (98.2–211.0)	0.04
d-Dimer, mg/L	2.97 (0.36–21.00)	11.92 (4.25–18.19)	0.04	6.45 (0.36–56.63)	14.43 (4.25–50.00)	0.13
Neutrophil, 10 × 10^9^/L	6.34 (3.57–16.66)	12.12 (3.44–20.03)	0.62	17.14 (3.94–50.34)	17.55 (7.71–23.03)	0.67
PCT, ng/ml	0.29 (0.08–1.91)	1.01 (0.05–88.34)	0.89	2.58 (0.14–47.53)	3.37 (0.11–88.34)	0.92
ALT, IU/L	27 (4–110)	52 (34–1,204)	0.20	107 (4–1,000)	128 (40–1,400)	0.46
AST, IU/L	38.4 (9.2–92.6)	55.9 (48.0–1,487.0)	0.04	123 (26–800)	687 (53–1,568)	0.07

CK-MB, creatine kinase-MB; CK, creatine kinase; hsTnI, hypersensitive troponin I; NA, not available; BNP, brain natriuretic peptide; IL-6, interleukin-6; hsCRP, hypersensitive C reaction protein; PCT, procalcitonin; ALT, alanine aminotransferase; AST, aspartate aminotransferase.

Maximum values of laboratory tests during hospitalization were compared in the two groups (Table 2). Patients with neutrophil infiltration had a significantly higher median peak value of the inflammatory indicator hsCRP than those without neutrophil infiltration (134.0 mg/L, range 98.2–211.0 mg/L vs. 10.0 mg/L, range 9.5–163.5 mg/L; *p* = 0.04). Higher median peak AST was also evident in patients with neutrophil infiltration than in those without neutrophil infiltration, though the difference was not significant (687 IU/L, range 53–1568 IU/L vs. 123 IU/L, range 26–800 IU/L; *p* = 0.07). The median maximum value of CK-MB during hospitalization in neutrophil-infiltrated patients was significantly higher than that in patients without neutrophil infiltration (280.0 IU/L, range 14.0–996.0 IU/L vs. 38.7 IU/L, range 5.9–234.7 IU/L; *p* = 0.04) (Figure 3). Patients with neutrophil infiltration had higher median peak hsTnI during hospitalization than those without neutrophil infiltration, though the difference was not significant (1.112 ng/ml, range 0.008–7.775 ng/ml vs. 0.220 ng/ml, range 0.008–8.749 ng/ml; *p* = 0.56). Other main laboratory parameters were comparable in the two groups (Table 2). Heart failure occurred in one of the four patients with neutrophil infiltration, and atrial fibrillation occurred in six of the patients without neutrophil infiltration.

## Discussion

Since the global COVID-19 pandemic began, a considerable proportion of patients with COVID-19 have developed critical illnesses, and many have experienced multiple organ failures including the lung, heart, and other organs (14, 15). To investigate specific pathological changes in the heart, we conducted the present autopsy study of hearts of 26 critically ill patients who died of COVID-19 in Wuhan from February 2020 to April 2020. The main findings of the study were that (1) active myocarditis was commonly and specifically accompanied by neutrophil infiltration; (2) the positive IHC detection rates of TNF-α and IL-6 were significantly higher in patients with neutrophil infiltration than in those without neutrophil infiltration, but this was not associated with the extent of lymphocyte or macrophage infiltration; (3) in patients with neutrophil infiltration, the time from syndrome onset to hospitalization was significantly longer than it was in those without neutrophil infiltration, and they exhibited higher baseline levels of CK, AST, hsCRP, and D dimer; (4) patients with neutrophil infiltration had significantly higher levels of CK-MB and non-significantly higher levels of hsTnI than those without neutrophil infiltration.

### Role of neutrophil: A new notion of COVID-19-related myocarditis?

Compared to the dramatic pathological changes described in the lung (16, 17), microscopic findings derived from the heart in patients with COVID-19 are less numerous and less specific. Previous post-mortem studies have identified various pathological manifestations, most pertaining to necrosis, myocarditis, inflammatory infiltration, and fibrin microthrombi (3, 4, 18). Compared with other published autopsy studies, the scattered necrotic cardiomyocytes in the current study were more common, probably because all the patients had severe COVID-19 and significantly longer ICU stays, which greatly increased the risks of cardiac injury. Consistent with previous reports, a small proportion of patients had active myocarditis, indicating more severe cardiac injury (19). Surprisingly neutrophil infiltration was detected in all four patients with myocarditis in the present study, but it was rare in patients without myocarditis. A series of trials indicate that neutrophil infiltration into pulmonary tissues causes the deterioration of patients with COVID-19 (20–23). Infiltrating neutrophils may release neutrophil extracellular traps (NETs)—which are extracellular networks of chromatin and microbicidal proteins—in response to SARS-CoV-2 infection, while excessive activation of NETs simultaneously results in lung cell death in critically ill patients (24–27). NETs derived from neutrophils are responsible for multiple pathophysiological changes including microthrombi, angiotensin-converting enzyme 2 activity, and oxidative stress (28, 29). Moreover, NETs are reportedly correlated with cytokine storms. Various cytokines may mediate the migration of neutrophils to injury sites (30). Conversely, the generation of NETs may stimulate the aggravation of cytokine storms including IL-6 via IL-1β (20, 31, 32). In the present post-mortem study, the positive detection rates of IL-6 and TNF-α were significantly higher in the four patients with neutrophil infiltration than in the 22 patients without neutrophil infiltration. Neutrophils, as the first-line regulator of adaptive immunity, are detrimental in cases of cardiac injury (33). Accumulating evidence indicates that autoimmune mechanisms may contribute to the progression of COVID-19 (34, 35). Based on our findings, despite a lack of direct evidence, it is reasonable to speculate that neutrophil infiltration may severely exacerbate cardiac injury in patients with severe COVID-19 by regulating autoimmune responses. Although the identification of NETs and autoimmunity was not part of the present study, results from the study indirectly indicate a strong association between neutrophils and severe cardiac injury.

Of the 26 patients in the current study, SARS-CoV-2 nucleic acids were only found in the heart tissues of five patients via real-time RT-PCR, as reported previously (12). Interestingly, none of these five patients had pathologically diagnosed myocarditis (36). Also, other pathological studies from endomyocardial biopsy (EMB) or autopsy rarely reported direct invasion of SARS-CoV-2 into cardiomyocytes (37). Current evidence still fail to determine the key role of SARS-CoV-2 infection on cardiac injury, while inflammatory infiltration was now regarded as a preliminary cause of heart damage in severe COVID-19. The Dallas Criteria (38) which only depends on histological evidence have already been not fully suitable for diagnosis of myocarditis, and IHC analysis for inflammatory infiltration was particularly advocated (39). A comparison of CD3^+^ T cells and CD68^+^ macrophages in patients with COVID-19 and control patients was reported in a recent review (37). There were no significant differences in the total numbers of CD3^+^ or CD68^+^ cells in the two groups, whereas CD68^+^ cell counts were significantly higher in the COVID-19 group than in the control group (37). In the present study, CD68^+^ macrophages were single or clustered in the myocardium, but there was no significant difference in the numbers of CD68^+^ cells in patients with and without active myocarditis. Moreover, all patients with myocarditis exhibited neutrophil infiltration accompanied by distinct cytokines, which was not prevalent in patients without myocarditis. Therefore, based on previous investigations of associations between neutrophil infiltration and critical COVID-19, we surmise that neutrophils and inflammatory infiltration may be a constituent cause of devastating heart damage in cases involving myocarditis. Some researchers have postulated that glucocorticoids may act as an immunomodulator that inhibits cytokine storms and excessive immune responses, improving therapeutic effects in critically ill patients (40, 41). Other specific cytokine inhibitors such as the IL-6 receptor inhibitor tocilizumab are being investigated (42, 43). The role of neutrophil infiltration in severe cardiac injury in patients with COVID-19 warrants further attention.

### Cardiac biomarkers: A warning sign of severe COVID-19?

In the present study, patients with cardiac neutrophil infiltration had a significantly longer median ICU stay than patients without neutrophil infiltration, indicating that cardiac neutrophil infiltration may be related to severe COVID-19. Several studies evaluating risk factors for a poor COVID-19 prognosis have identified a series of laboratory predictors of in-hospital mortality, including AST, D dimer, and hsCRP (44, 45). Significant elevation of baseline levels of these three parameters was also found in patients with cardiac neutrophil infiltration in the current study. There may be a link between a relatively severe condition (involving liver function, coagulation, and inflammation) and pathological changes in heart tissues, indicating that COVID-19 can simultaneously cause damage to multiple organs that are not part of the respiratory system. Of the patients in the present study, however, almost half were not tested for cardiac biomarkers until they were transferred to the ICU or exhibited relevant symptoms. This suggests that under an emergent situation, inspection of the heart may be easily neglected by physicians, who mainly focused on treatment strategies based on the respiratory system. Furthermore, patients with neutrophil infiltration exhibited relatively worse conditions both on admission and during their ICU stay. The peak level of CK-MB throughout hospitalization was significantly higher in patients with neutrophil infiltration than in those without neutrophil infiltration. Peak hsTnI was also higher in patients with neutrophil infiltration, although this observation was not statistically significant due to the small sample size. The dramatic elevation of cardiac biomarkers was consistent with severe pathological changes in hearts. In combination with indicators identified in previous studies (1, 2), biomarkers of cardiac injury including CK-MB and troponin may be indicators of heart damage, and predictors of a systemic inflammatory response to COVID-19. A series of observational cohort studies conclusively indicate that CK-MB and other cardiac injury biomarkers are independent predictors of ICU admission and fatality in patients with COVID-19, regardless of the presence or absence of comorbid coronary artery disease (46–48). The current study in combination with previous clinical evidence collectively demonstrates that cardiac biomarkers, as meaningful indicators of critical illness, should be paid particular attention by clinical physicians in patients with COVID-19.

The present study had several limitations. The sample size was low, and findings from 26 patients inevitably entail potential bias, particularly with respect to the association between cardiac injury parameters and neutrophil infiltration. Second, although patients with neutrophil infiltration in hearts had significantly longer ICU stays than patients without, we could not conclude that neutrophil infiltration may prolong the ICU stay because all patients ultimately died, rather than recovering. Further studies may focus on this issue and qualify neutrophil infiltration in hearts as a predictor of prognosis in patients with severe COVID-19. Third, IHC staining for NETs was not performed, though the effects of NETs on COVID-19 have been investigated in previous studies. Lastly, we did not investigate details of heart injury mechanisms due to COVID-19 further, and more research is needed in this regard.

In this autopsy study of heart tissue from critically ill patients who died of COVID-19, active myocarditis was commonly accompanied by neutrophil infiltration. Patients with neutrophil-infiltrated myocarditis had more severe abnormal baseline laboratory test results for AST, D dimer, and hsCRP, and a higher peak value of CK-MB during hospitalization than patients without neutrophil-infiltrated myocarditis. The role of neutrophils in severe heart injury and systemic conditions in COVID-19 should be emphasized.

## Data availability statement

The raw data supporting the conclusions of this article will be made available by the authors, without undue reservation.

## Ethics statement

The studies involving human participants were reviewed and approved by National Health Commission of China and the Helsinki Declaration. The patients/participants provided their written informed consent to participate in this study.

## Author contributions

QZ collected the specimens and designed the conduction of study. HZ and XYao were responsible for specimen disposal and pathological analysis of all cases. XYan collected and analyzed the clinical information of recruited patients. SM was in charge of statistical analysis and manuscript writing. QZ and SM verified the underlying data. YS, YP, MC, CP, SW, ML, and CY provided assistance of staining procedure and figure exhibition. XB, YH, and SZ contributed to the leadership of the whole process of study conduction, and acted as the key role of initiating, designing, conducting, and concluding the study. All authors had full access to all the data in the study and accept responsibility to submit for publication.

## Funding

Supported by the Emergency Key Program of Guangzhou Laboratory (EKPG21-32), National Science Funding of China (NSFC 32071116), and LIAONING S&T Project (2020JH1/10300002).

## Conflict of interest

The authors declare that the research was conducted in the absence of any commercial or financial relationships that could be construed as a potential conflict of interest.

## Publisher's note

All claims expressed in this article are solely those of the authors and do not necessarily represent those of their affiliated organizations, or those of the publisher, the editors and the reviewers. Any product that may be evaluated in this article, or claim that may be made by its manufacturer, is not guaranteed or endorsed by the publisher.
