# Supplementary material for: Neutrophil infiltration and myocarditis in patients with severe COVID-19: A post-mortem study
Source: Front Cardiovasc Med. 2022 Oct 14;9:1026866. doi: 10.3389/fcvm.2022.1026866 (PMC9614157; doi:10.3389/fcvm.2022.1026866)
Supplement: Supplementary file 1 [file Data_Sheet_1.docx]

**Supplemental Materials**

**Table S1. Baseline Patient Characteristics**

|  | **Total (n=26)** |
| --- | --- |
| **Age, years, median (range)** | 68 (53-88) |
| **Male, No. (%)** | 13 (50%) |
| **Comorbidities, No. (%)** |  |
| Hypertension | 9 (35%) |
| Cardiovascular disease | 10 (39%) |
| Diabetes | 4 (15%) |
| Chronic pulmonary disease | 6 (23%) |
| **Complications, No. (%)** |  |
| DIC | 7 (27%) |
| Heart failure | 10 (39%) |
| Renal dysfunction | 13(50%) |
| Anemia | 18 (69%) |
| **Medications during hospitalization, No. (%)** |  |
| Antibiotics | 26 (100%) |
| Chinese herbs | 12 (46%) |
| Gamma globulin | 13 (52%, available on 25 patients) |
| Antiplatelet therapy | 2 (8%, available on 24 patients) |
| Anticoagulation | 18 (69%) |
| GC | 20 (77%) |
| **Procedures during hospitalization, No. (%)** |  |
| Non-end-stage endotracheal intubation | 16 (62%) |
| Mechanical ventilation | 22 (85%) |
| ECMO | 6 (23%) |
| Blood transfusion | 11 (42%) |
| Bronchoscopy | 7 (27%) |
| Dialysis | 6 (23%) |

DIC: disseminated intravascular coagulation; GC: glucocorticoid; ECMO: extracorporeal membrane oxygenation.

**Figure S1. Other various types of pathologic findings besides myocarditis**

**
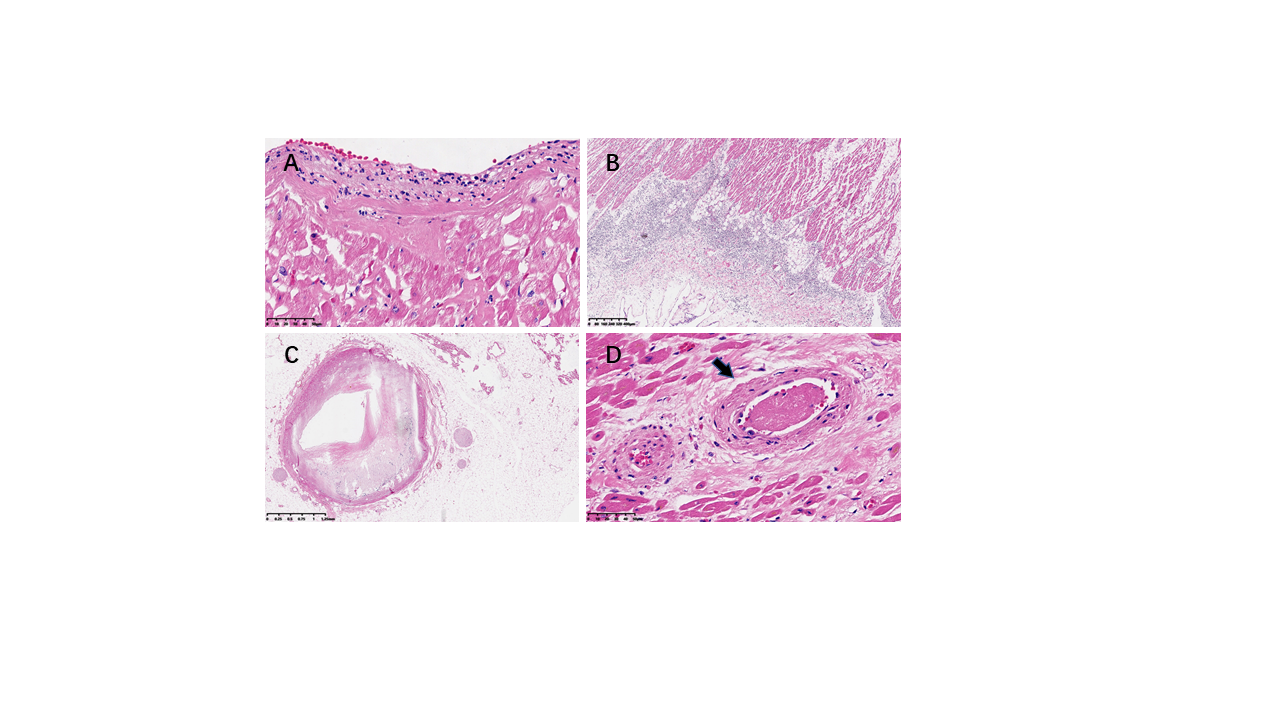
**

Figure S1 shows representative various types of pathologic findings besides myocarditis. A) endocarditis in a 62-year-old man with myocarditis; B) epicarditis in a 56-year-old woman with myocarditis; C): atherosclerosis in a 64-year-old man without myocarditis; D): microthrombus in a 62-year-old man with myocarditis. The arrow denotes the small vascular microthrombus in myocardial interstitium. Scale bars represent 50μm.
